# IL-17-Expressing CD4^**+**^ and CD8^**+**^ T Lymphocytes in Human Toxoplasmosis

**DOI:** 10.1155/2014/573825

**Published:** 2014-08-17

**Authors:** Jéssica Líver Alves Silva, Karine Rezende-Oliveira, Marcos Vinicius da Silva, César Gómez-Hernández, Bethânea Crema Peghini, Neide Maria Silva, José Roberto Mineo, Virmondes Rodrigues Júnior

**Affiliations:** ^1^Course of Tropical Medicine and Infectology, Laboratory of Immunology, Federal University of Triângulo Mineiro, Avenida Frei Paulino, No. 30, 38025-180 Uberaba, MG, Brazil; ^2^Laboratory of Biomedical Sciences, Federal University of Uberlândia, Rua 20, No. 1600, 38304-402 Ituiutaba, MG, Brazil; ^3^Course of Tropical Medicine and Infectology, Laboratory of Parasitology, Federal University of Triângulo Mineiro, Avenida Frei Paulino, No. 30, 38025-180 Uberaba, MG, Brazil; ^4^Laboratory of Histology and Embryology, Federal University of Uberlândia, Avenida Pará, No. 1720, 38400-902 Uberlândia, MG, Brazil; ^5^Laboratory of Immunology, Federal University of Uberlândia, Avenida Pará, No. 1720, 38400-902 Uberlândia, MG, Brazil

## Abstract

This study aimed to measure the synthesis of Th1 and Th2 cytokines by mononuclear cells after culture with live *T. gondii* and identified Th17 (CD4^+^) and Tc17 (CD8^+^) cells in toxoplasma-seronegative and toxoplasma-seropositive parturient and nonpregnant women. Cytometric bead arrays were used to measure cytokine levels (IL-2, TNF-*α*, IFN-*γ*, IL-4, IL-5, and IL-10); immunophenotyping was used to characterize Th17 and Tc17 cells, and the cells were stained with antibodies against CD4^+^ and CD8^+^ T cells expressing IL-17. The addition of tachyzoites to cell cultures induced the synthesis of IL-5, IL-10, and TNF-*α* by cells from seronegative parturient women and of IL-5 and IL-10 by cells from seropositive, nonpregnant women. We observed a lower level of IL-17-expressing CD4^+^ and CD8^+^ T lymphocytes in cultures of cells from seronegative and seropositive parturient and nonpregnant women that were stimulated with tachyzoites, whereas analysis of the CD4^+^ and CD8^+^ T cell populations showed a higher level of CD4^+^ T cells compared with CD8^+^ T cells. These results suggest that the cytokine pattern and IL-17-expressing CD4^+^ and CD8^+^ T lymphocytes may have important roles in the inflammatory response to *T. gondii*, thus contributing to the maintenance of pregnancy and control of parasite invasion and replication.

## 1. Introduction

*Toxoplasma gondii* is an obligate intracellular protozoan that causes toxoplasmosis [1], which is an opportunistic infection that may manifest during the immunosuppression/immunodepression process of the infected patient [2]. Toxoplasmosis is considered to be a disease of major clinical importance to pregnant women [3] because the parasite can be transmitted through the placenta and cause abortion or fetal malformation [4].

The main mechanism of elimination of intracellular parasites is the host Th1 immune response, which results in the production of cytokines such as interleukin-2 (IL-2), interferon-gamma (IFN-*γ*), tumor necrosis factor-alpha (TNF-*α*), and interleukin-12 (IL-12) [5]. Th17 cells, another subset of effector T cells, produce the cytokines IL-17, IL-21, and IL-22 [6, 7] and contribute to the inflammatory response during parasite infection. IL-23 is produced by cells of the innate immune system [6] and is able to induce the generation and expansion of IL-17-producing T cells [8].

Cytotoxic CD8^+^ T cells are essential for the development of specific immune responses and may be differentiated into effector subtypes that are characterized by their cytokine expression profiles after stimulation by antigens. A different subset of CD8^+^ cells has been related to this process, but one type, known as Tc17, can express IL-17 in the presence or absence of IFN-*γ* and IL-4. Phenotypic analysis shows that Tc17 cells do not express perforin or granzyme B, and they are not capable of mediating a significant* in vitro* cell lysis process, regardless of the production of their standard cytokines [9].

Therefore, it is believed that Tc17 cells have a different chemotactic activity than that observed in Th17 cells; therefore, they may migrate to inflammatory sites in response to different chemokines [10] and can be defined as a different subset of CD8^+^ T cells with their own phenotypic and functional characteristics [11].

It is known that maternal tolerance to fetal alloantigens is characterized by the response of regulatory T cells, which are prevalent during pregnancy, that produce cytokines such as IL-4, IL-5, IL-10, and TGF-*β* [12–14]. Although the inflammatory process is necessary for successful implantation, a severe inflammatory process may lead to embryo reabsorption. Cells that produce/express IL-17 are involved in this process; therefore, this cytokine may influence the pathophysiology of premature labor [15].

Taking into account the modulation characteristics of Th17/Tc17 cells, particularly their role in different inflammatory-infectious processes and the possible influences they may have on pregnancy stability, this study aimed to evaluate IL-17-expressing CD4^+^ and CD8^+^ T lymphocytes and the levels of cytokines synthesized by mononuclear cells of the peripheral blood of parturient and nonpregnant women that had been characterized as seropositive or seronegative for* T. gondii* infection.

## 2. Materials and Methods

### 2.1. Subjects

Sixty-two women aged 20–45 years were enrolled in the present study. Among them, 14 were parturient with negative serology for* Toxoplasma gondii*, 23 were parturient and seropositive for anti-*T. gondii* antibodies, 16 were nonpregnant women who were seronegative for* T. gondii*, and 9 were nonpregnant women who were seropositive for anti-*T. gondii* antibodies. The parturient patients (3rd trimester of pregnancy) were recruited from the Outpatient Clinic of Obstetrics at the General Hospital of Federal University of Triângulo Mineiro (UFTM). Students and Immunology Laboratory staff were invited to join the nonpregnant group. The volunteers did not have other inflammatory diseases and/or acute or chronic infections, and the parturient volunteers had no complications during pregnancy and did not use any medicine. The serology for IgG and IgM anti-*T. gondii* antibodies from each volunteer was determined using chemiluminescence (Biomerieux, São Paulo, SP, Brazil), and parturient and nonpregnant women who were serologically positive for IgM anti-*T. gondii *antibodies were excluded from the study.

All volunteers signed a consent form to take part in the study. This research project was approved by the Research Ethics Committee of Federal University of Triângulo Mineiro, Brazil, under protocol number 1348 in 2009 and was conducted in accordance with the Declaration of Helsinki (1964).

### 2.2. Peripheral Blood Mononuclear Cells (PBMCs) of the Patients

To obtain cell suspensions, 10 mL of peripheral blood samples was collected in heparinized tubes (Vacutainer). Mononuclear cells were isolated using Ficoll-Hypaque (density: 1.074) (Invitrogen, Grand Island, New York, USA) followed by centrifugation at 400 ×g for 25 minutes at 25°C. The mononuclear cell band was subjected to a washing procedure with sterile RPMI-1640 medium (Gibco BRL, Grand Island, USA) at 400 ×g for 10 minutes at 8°C. After washing, these cells were resuspended in RPMI-1640 culture medium supplemented with 10% fetal bovine serum (FBS) (Gibco BRL), 50 mM Hepes (Gibco BRL), and 40 *μ*L/mL Gentamicin (Schering-Plough, São Paulo, SP, Brazil) (complete RPMI). The cells were counted in a Neubauer chamber, and the final concentration was adjusted to 5 × 10^5^ cells/mL. Cell viability was determined by 0.2% Trypan blue exclusion. Cell suspensions with viability > 90% were used in both cell culture and immunophenotyping experiments.

### 2.3. Preparation of* In Vitro* Tachyzoites

Human foreskin fibroblast (HFF) cells were cultivated in RPMI-1640 culture medium (Gibco BRL) supplemented with 5% heat-inactivated fetal bovine serum (FBS) (Gibco BRL) and 40 mg/L Garamycin (Schering-Plough). Subsequently, the cells were incubated at 37°C in a CO_2_ incubator. Live tachyzoites of the low-virulence ME49 strain of* T. gondii* were used. These parasites were kept in continuous culture in confluent monolayers of HFF cells that were then infected at a ratio of 3 tachyzoites/cell. After lysis of the infected cells, the culture supernatant was collected and subjected to centrifugation at 400 ×g for 10 minutes. The sediment was suspended in supplemented RPMI-1640 medium to obtain enriched preparations of tachyzoites.

### 2.4. Detection of IL-17-Expressing CD4**^+^** T and CD8**^+^** T Cells

After cell concentration adjustment in a Neubauer chamber (5 × 10^5^ cells/mL), the cells were seeded into a 96-well plate (Sarstedt, Newton, North Carolina, USA) for culturing and later identification of IL-17-producing CD4^+^ T and CD8^+^ T cells.

Mononuclear cells in the culture of each subject were subjected to three conditions, as follows.

*(1) Unstimulated PBMCs (Negative Control)*. Complete RPMI-1640 medium is in a final volume of 200 *μ*L/well.

*(2) Nonspecific Stimulation with Anti-CD3* ^*±*^ *(Positive Control).* 50 *μ*L of anti-CD3 (1 mg/mL) (mouse anti-human CD3, BD Pharmingen, San Diego, USA) was added to the culture plate and incubated for 40 minutes to allow for adhesion to the plate.

*(3) Specific Stimulation with Live T. gondii Tachyzoites (ME49 Strain)*. The concentration of parasites used to infect mononuclear cell cultures (1 × 10^6^ parasites/well) was adjusted after counting the cells in a Neubauer chamber.

The plates were incubated in a 5% CO_2_ incubator at 37°C, and after 48 hours of culture, we added 100 mL of 10% RPMI-1640 complete medium into each well of the plate. Six hours before the end of the culture, we added 1 *μ*L GolgiStop (BD Biosciences) into the wells submitted to culture conditions 2 and 3.

The supernatant was collected for later cytokine detection. The cells that remained in the wells were suspended in the plate in 1× phosphate-buffered saline (PBS), collected, and placed in cytometry tubes (BD Biosciences). Then, a PBS/skim milk solution was added to the tubes (to block nonspecific interactions), which were incubated for 20 minutes. Subsequently, 4 *μ*L of CD4 mouse anti-human mAb conjugated to phycoerythrin (CD4^+^-PE) (BD Pharmingen San Diego, USA) and 4 *μ*L CD8^+^ mouse anti-human mAb conjugated to CD8-peridinin chlorophyll protein (PerCP) (BD Pharmingen) were added to two tubes. These tubes were stored in a dark chamber at room temperature for 20 minutes. Next, the cell solutions were washed, and 100 *μ*L of a cell fixative and permeabilizing solution (BD Cytofix/Cytoperm Plus Fixation Kit, BD Pharmingen) was added to the pellets of each tube. Then, the solutions were incubated in a dark chamber at room temperature for 20 minutes. After incubation, the tubes were washed again, the supernatants were discarded, and 100 *μ*L of permeabilizing solution (BD Cytofix/Cytoperm Plus Fixation Kit, BD Pharmingen) was added to the pellets of each tube, followed by the addition of 3 *μ*L intracellular anti-IL-17 (BD Pharmingen) and staining with fluorochrome Alexa Fluor 488. All tubes were incubated in a dark chamber at room temperature for 20 additional minutes. Finally, the cells were washed with an appropriate buffer solution, and 1% PBS/formaldehyde was added to the tube.

The cells were obtained by flow cytometry using a BD FACSCalibur (Becton & Dickinson) and analyzed using the CellQuest software. To quantify the T lymphocytes, we analyzed the forward-scattered light (FSC) and side-scattered light (SSC) parameters, and the cells in the mononuclear cell gate were selected and analyzed for CD4 and CD8 expression in the CD4^+^ and CD8^+^ T cell populations. This analysis allowed for the collection of statistical data on the cells in the corresponding quadrants, where a new gate was established to analyze the expression of IL-17 by these cells.

### 2.5. Quantification of the Cytokines in the Culture Supernatants

Quantification of the cytokines IL-2, IL-4, IL-5, IL-10, IFN-*γ*, and TNF-*α* in the culture supernatants was performed using a cytometric bead array (CBA, BD Pharmingen). The supernatants were incubated in cytometry tubes containing microspheres conjugated to capture and detect antibodies for each phycoerythrin-conjugated cytokine, and sandwich complexes were formed. A standard curve was concomitantly generated for each cytokine submitted to the assay. After incubation, analyses using a FACSCalibur (Becton & Dickson) and FCAP Array software were performed. The PE fluorescence intensity of each complex point determined the concentration of each cytokine in comparison with the standard curve. The data were calculated and expressed in pg/mL. The sensitivity ranged from 1.37 pg/mL to 12.500 pg/mL.

### 2.6. Statistical Analysis

The data were subjected to normality and homogeneity tests. For statistical analysis, STATVIEW software was used to perform Wilcoxon tests to evaluate the variation within the same group, the Mann-Whitney test was used to evaluate the differences between two groups, and the Kruskal-Wallis test was used to evaluate differences between four groups. Differences were considered statistically significant when *P* < 0.05.

## 3. Results

### 3.1. Expression of Th1 and Th2 Cytokines Produced by Mononuclear Cells of Parturient and Nonpregnant Women Who Were Seropositive or Seronegative for Anti-*T. gondii *Antibodies

The production of Th1 and Th2 cytokines was analyzed in culture supernatants of mononuclear cells of peripheral blood in the absence of stimuli or in the presence of* T. gondii* tachyzoites. Analyses were performed for each group taking into account, at first, the pregnancy parameter and then previous exposure to* T. gondii.*

**Figure 1:**
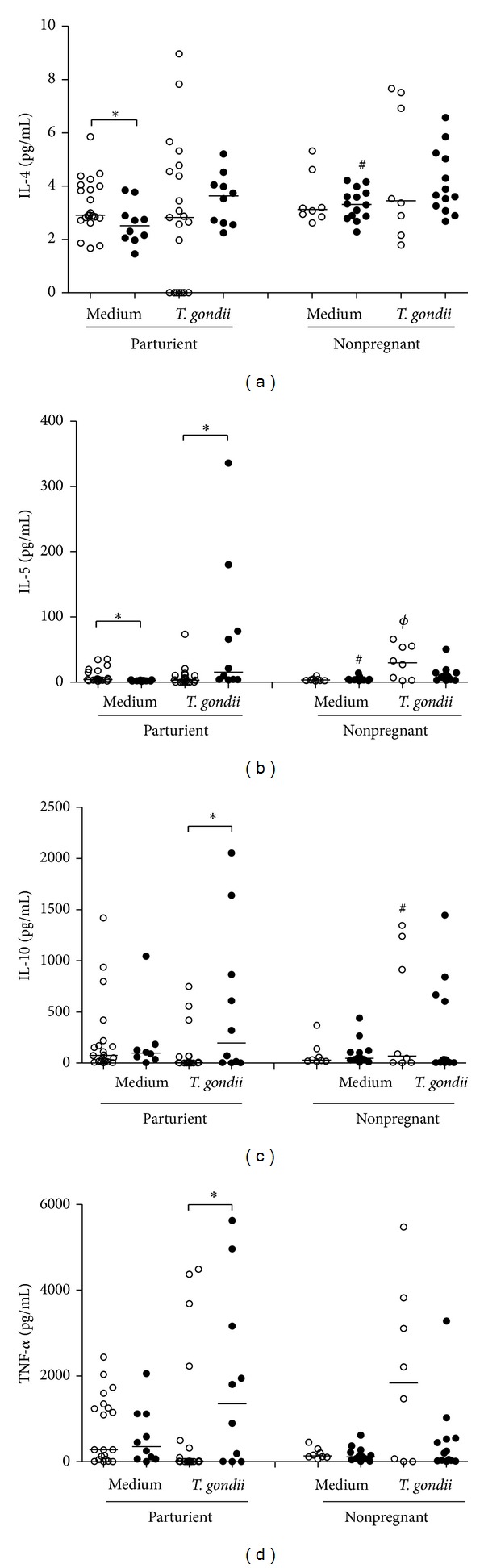
Cytokine production by PBMCs in culture supernatants stimulated with* T. gondii*. The horizontal line indicates the median, open circles indicate the* T. gondii* seronegative subjects, and filled circles indicate seropositive subjects. (a) *IL-4 expression from cells of seronegative and seropositive parturient women without the stimulus; ^#^IL-4 expression from cells of seronegative parturient and nonpregnant women without the stimulus; (b) *IL-5 expression from cells of seronegative and seropositive parturient women without the stimulus and after stimulation with live tachyzoites; ^#^IL-5 expression from cells of seronegative parturient or nonpregnant women without the stimulus; ^*ϕ*^IL-5 expression from cells of seropositive parturient or nonpregnant women after stimulation with live tachyzoites; (c) *IL-10 expression from cells of seronegative or seropositive parturient women after stimulation with live tachyzoites; ^#^IL-10 expression from cells of seropositive parturient or nonpregnant women after stimulation with live tachyzoites; (d) *TNF-*α* expression from cells of seropositive or seronegative parturient women after stimulation with live tachyzoites. (*P < 0.05, Wilcoxon test). ^#,*ϕ*^Significant differences between the PMBCs of seropositive nonpregnant and parturient women stimulated with the* T. gondii* strain (*P* < 0.05, Mann-Whitney test).

As shown in Figure 1, the addition of live* T. gondii* tachyzoites to cell cultures of seronegative parturient women induced positive modulation of the synthesis of IL-5, IL-10, and TNF-*α* (*P* = 0.012, 0.033, and 0.050, resp.) by these cells, and these levels were compared with those produced by cells of seropositive parturient women. Moreover, there was a significant increase in the production of both IL-4 (*P* = 0.044) and IL-5 (*P* = 0.001) by cells of seropositive parturient women in comparison with seronegative parturient women in unstimulated cultures. Furthermore, in unstimulated cultures, the cells of seronegative nonpregnant women produced significant levels of IL-4 (*P* = 0.024) and IL-5 (*P* = 0.001) in comparison with the cells of seronegative parturient women. The detected IL-4 levels were lower than those of other cytokines (Figures 1(a) and 1(b)).

The addition of* T. gondii* tachyzoites to cell cultures of seropositive parturient and nonpregnant women led to increased synthesis of IL-5 (*P* = 0.015) and IL-10 (*P* = 0.031) by these cells in relation to the cells of seropositive parturient women.

We did not observe a significant difference between the groups when the syntheses of IFN-*γ* and IL-2 were analyzed (data not shown).

### 3.2. Cultured IL-17-Expressing CD4^+^ or CD8^+^ T Cells of Parturient or Nonpregnant Patients in the Presence or Absence of Live* T. gondii* Tachyzoites

**Figure 2:**
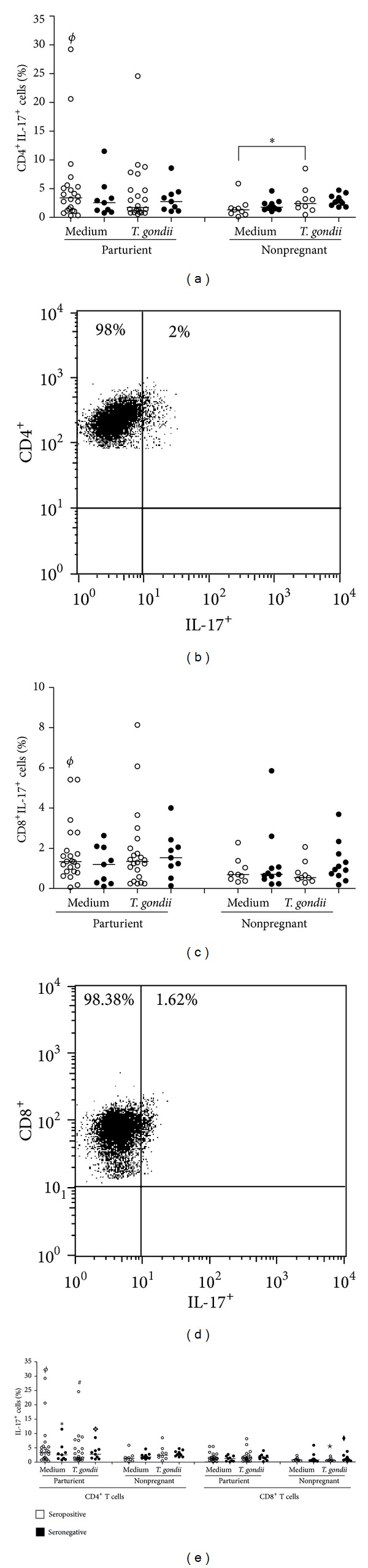
IL-17^+^CD4^+^ T cells and IL-17^+^CD8^+^ T cells from nonpregnant or parturient women. The horizontal line indicates the median, open circles indicate the* T. gondii* seronegative subjects, and filled circle indicate seropositive subjects. (a) The percentage of CD4^+^ T cells expressing IL-17. (c) The percentage of CD8^+^ T cells expressing IL-17. (e) Comparison CD4^+^ T cells and CD8^+^ T cell expressing IL-17. One representative dot-plot from each is shown ((b)—seropositive, nonpregnant, and unstimulated ×* T. gondii* stimulated), ((d)—seropositive, parturient, unstimulated × seropositive, nonpregnant, and unstimulated). Gated on CD4^+^ and CD8^+^ T cells. The horizontal line indicates the median, bars indicate the 25% and 75% percentiles, and vertical lines indicate the 10% and 90% percentiles. (*P < 0.05, Wilcoxon test). ^*❖*,★,◆,*ϕ*^Significant differences between PMBCs of seropositive, nonpregnant and parturient women stimulated with* T. gondii* strain (*P* < 0.05, Mann-Whitney test).

As shown in Figure 2, PBMC cultures infected with live tachyzoites from seropositive nonpregnant patients had significantly higher levels of IL17-expressing CD4^+^ T cells than unstimulated cultures (*P* = 0.05). The comparison of unstimulated cell cultures of seropositive parturient women in relation to those of nonpregnant women allowed us to observe a higher level of CD4^+^ T cells expressing IL-17 in cultures from parturient women (*P* = 0.045) (Figures 2(a) and 2(b)).

CD8^+^ T cell cultures from parturient women who were seropositive for anti-*T. gondii* antibodies that had not been subjected to stimuli had significant expression (*P* = 0.05) of IL-17 in comparison with nonpregnant women with the same serology (Figures 2(c)  and 2(d)).

When comparing the IL-17 expression by CD4^+^ T and CD8^+^ T cells when live* T. gondii* tachyzoites were added or not added to the PBMC cultures of seropositive or seronegative parturient patients (*P* = 0.0002 and *P* = 0.006, resp.), a higher number of CD4^+^ T cells than CD8^+^ T cells expressing IL-17 were observed. After stimulation of PBMC cultures from nonpregnant women with live* T. gondii* tachyzoites, we observed a higher level of CD4^+^ T cells than CD8^+^ T cells expressing IL-17, regardless of the serology of the patients (*P* = 0.007) (Figure 2(e)).

## 4. Discussion

During pregnancy, cytokine production by maternal cells may vary according to the trimester of pregnancy [15]. Due to this variation in cytokine production, parturient women may become immunologically vulnerable and may be stricken by diseases such as toxoplasmosis, which can be transmitted to the fetus during primary infection [16].

The present study showed that mononuclear cells of seronegative parturient women cultured with* T. gondii* tachyzoites produced significant levels of IL-5, IL-10, and TNF-*α* compared with the cells of seropositive parturient women. This work demonstrated the Th2 cytokine production after challenge by a nonvirulent strain of* T. gondii*, which makes this study distinct from the other by our group, where we analyzed the behavior and antigenic capacity of two distinct strains of* T. gondii* (a virulent and a nonvirulent strain) to stimulate the production of Th1 cytokines [17].

We noticed that the addition of tachyzoites to the culture led to a significant increase in the levels of TNF-*α* that were produced by cells of seronegative parturient women in relation to seropositive parturient women [17]. In another study, cells of seronegative nonpregnant women were shown to synthesize significant levels of IL-10 after the addition of tachyzoites to the culture. The present assay revealed that the addition of live parasites to the culture induced an increase in IL-10 synthesis by cells of seropositive nonpregnant women, which is an outcome that differs from the findings of our previous study. IL-10 has proved to be important in the preservation of tissue integrity in several experimental models of infectious diseases, including toxoplasmosis [18].

Matowicka-Karna et al. [19, 20] showed that nonpregnant women who were seropositive for* T. gondii* antibodies had significantly higher levels of IL-5 than seronegative women. Similar results were demonstrated in our study, which showed that cells from seropositive parturient women produced higher levels of IL-5 than those from seronegative parturient women. However, when the cell culture was infected with live tachyzoites, the IL-5 levels were reduced, suggesting that parasitic mechanisms may influence cytokine production.

The lower amounts of detected IL-4 may be related to gestational age (parturient), which is characterized by a proinflammatory or period of cell culture (nonpregnant). Even though they were at low levels, we observed increased IL-4 in the supernatant of seropositive parturient cells compared with seronegative cells, suggesting that the presence of a parasite can modulate cytokine synthesis, resulting in increased susceptibility to* T. gondii* infection.

The levels of IL-17 were not measured due to the period that the cells were maintained in culture when conducting this study.

In the present study, we found a higher level of CD4^+^ T cells than CD8^+^ T cells expressing IL-17 (Th17) in cell cultures of parturient and nonpregnant women, regardless of their serology status for anti-*T. gondii* antibodies. The analysis of IL-17 synthesis by CD8^+^ and CD4^+^ T cells in healthy adult individuals demonstrates that the level of IL-17-producing CD8^+^ T cells was lower than that of CD4^+^ T cells [21]. During pregnancy, significant amounts of IL-17-producing CD4^+^ T cells are present, and there is no variation in the number of these cells between the second and third trimesters of pregnancy [21, 22].

The percentage of CD4^+^ T cells that expressed IL-17 is significantly higher in the placenta of pregnant mice infected with* T. gondii* than in control mice [23]. In this study, we observed that there was a higher level of CD4^+^ and CD8^+^ T cells expressing IL-17 in PBMC cultures of seropositive parturient women who were infected with live* T. gondii* tachyzoites than in the unstimulated culture.

PBMC cultures of nonpregnant women who were seropositive for anti-*T. gondii* antibodies significantly expressed IL-17 by CD4^+^ T cells (Th17) in cultures stimulated with live* T. gondii* tachyzoites, which is in consonance with results from Kelly et al. [24], who found that mice infected with* T. gondii* are capable of inducing a significant immune response, which is stimulated by IL-17, against parasites in the early stages of infection.

Furthermore, PBMC cultures of parturient and nonpregnant women, regardless of their serology, had a significant number of CD8^+^ T cells expressing IL-17. Few studies have investigated the main functions in this cellular phenotype [25].

This is the first study about a repertoire of IL-17-expressing CD4^+^ and CD8^+^ T lymphocytes and the production of Th2 cytokines related to human pregnancy and* T. gondii* infection. Our results suggest that these cells and cytokines may display an important role in the inflammatory response by contributing to the process of pregnancy maintenance and control of parasite invasion and replication.
